# Gut microbiota in post-acute COVID-19 syndrome: not the end of the story

**DOI:** 10.3389/fmicb.2024.1500890

**Published:** 2024-12-24

**Authors:** Yaping An, Linlin He, Xin Xu, Meiyu Piao, Bangmao Wang, Tianyu Liu, Hailong Cao

**Affiliations:** Tianjin Key Laboratory of Digestive Diseases, Department of Gastroenterology and Hepatology, Tianjin Institute of Digestive Diseases, National Key Clinical Specialty, General Hospital, Tianjin Medical University, Tianjin, China

**Keywords:** coronavirus disease 2019, post-acute COVID-19 syndrome, severe acute respiratory syndrome coronavirus-2, gut microbiota, diet, gut-lung axis

## Abstract

The coronavirus disease 2019 (COVID-19), caused by severe acute respiratory syndrome coronavirus-2 (SARS-CoV-2), has led to major global health concern. However, the focus on immediate effects was assumed as the tip of iceberg due to the symptoms following acute infection, which was defined as post-acute COVID-19 syndrome (PACS). Gut microbiota alterations even after disease resolution and the gastrointestinal symptoms are the key features of PACS. Gut microbiota and derived metabolites disorders may play a crucial role in inflammatory and immune response after SARS-CoV-2 infection through the gut-lung axis. Diet is one of the modifiable factors closely related to gut microbiota and COVID-19. In this review, we described the reciprocal crosstalk between gut and lung, highlighting the participation of diet and gut microbiota in and after COVID-19 by destroying the gut barrier, perturbing the metabolism and regulating the immune system. Therefore, bolstering beneficial species by dietary supplements, probiotics or prebiotics and fecal microbiota transplantation (FMT) may be a novel avenue for COVID-19 and PACS prevention. This review provides a better understanding of the association between gut microbiota and the long-term consequences of COVID-19, which indicates modulating gut dysbiosis may be a potentiality for addressing this multifaceted condition.

## 1 Introduction

Since the pandemic of coronavirus disease 2019 (COVID-19) caused by severe acute respiratory syndrome coronavirus 2 (SARS-CoV-2) (Sungnak et al., 2020; Zhu et al., 2020), multiple variants have been identified, which act as a threat to public health and have been speculated to coexist with humans for a long time (Kissler et al., 2020; Kim, 2024). The clinical manifestations of COVID-19 mostly focus on the respiratory systems, including fever, cough and dyspnea, pneumonia, acute respiratory distress syndrome (ARDS), and respiratory failure (Chen N. et al., 2020). However, the symptoms may be multisystem and have long-lasting effects on people termed long-COVID or post-acute COVID-19 syndrome (PACS), which persist beyond 4 weeks after infection up to several years and involve physical, cognitive, and mental health impairments (Parotto et al., 2023; Santos et al., 2024). The prevalence of PACS is approximately 10–30% and over 65 million individuals worldwide are plagued (Davis et al., 2023). More than one-fifth of COVID-19 adult patients could not recover within 3 months, especially for women and those with cardiovascular disease (Oelsner et al., 2024). Among hospitalized COVID-19 individuals, the risk of PACS declined over the 3 years but still contributed 90 disability-adjusted life years (DALYs) per 1,000 persons, suggesting the burden of health loss remains even in the third year after infection (Cai et al., 2024). Due to the heterogeneity of disparate symptoms in long-COVID patients, different mechanisms have been proposed, including viral persistence or reactivation, permanent inflammation, immune dysregulation, and alteration of gut microbiota, but there is not an adequate perception of this complex condition (Fernández-de-Las-Peñas et al., 2023).

Numerous studies have revealed that dysregulation of gut microbiota is common in acute to post-COVID and has a significant impact on this process through the gut-lung axis. Direct or indirect microbial pathways are thought to contribute to the interaction between SARS-CoV-2 and the gut. Substantial evidences indicate that symbiotic microbiota regulates invading viruses through various mechanisms, which in turn are regulated by viruses, thereby exerting stimulatory or inhibitory effects on virus infections (Karst, 2016). Baseline gut microbiota and metabolome can modulate the durability of immunity to the SARS-CoV-2 vaccine to overcome waning immune responses over time and potentially predict immunogenicity to vaccines for up to 6 months (Peng et al., 2023). Additionally, the disordered gut microbiota is associated with persistent inflammation even after COVID-19 resolution, which suggests the long-reaching effect of the gut microbiota and hints at the significance of microbial management in long-COVID. Several studies have reported the detection of viral RNA in the feces and gastrointestinal tract of COVID-19 patients (Xing et al., 2020; Wu et al., 2020; Guo M. et al., 2021). The median duration of viral RNA in feces was found to be 22 days and the viral load typically peaked 2–3 weeks after symptom onset during the later stages of the disease (Zheng et al., 2020; Walsh et al., 2020). In some patients, fecal samples remained positive for the virus even when respiratory and/or sputum samples were negative (Xu et al., 2020; Wölfel et al., 2020).

While the development of pharmaceutical treatments for COVID-19 is currently in progress, the long-term effects of COVID-19 demand greater attention and coordinated preventive strategies. Nirmatrelvir-ritonavir was found to reduce the risk of post-acute inpatient death as well as cardiovascular and respiratory complications among hospitalized patients with COVID-19 (Wang H. et al., 2024). Pre-infection vaccination was associated with reduced risk of post-COVID conditions, including sensor, circulatory, blood and hematologic, skin and subcutaneous, and non-specific COVID-19 related disorders (Malden et al., 2024). Surprisingly, healthy lifestyle behaviors may play an important role in maintaining public health in the COVID-19 pandemic (Murakami et al., 2023). The causes of long-COVID are unclear, but certain dietary interventions may manage symptoms and support overall recovery (Cheong et al., 2023). The proper diet and supplementation are postulated to maintain the host homeostasis in COVID-19 and post-COVID by altering the microbial configuration, affecting host metabolism, and regulating the immune response. Individuals with a diet high in fat could reduce the richness and diversity of microbiota (Kolodziejczyk et al., 2019), which are highly prone to COVID-19 and related adverse outcomes (Moallemian et al., 2021). On the contrary, diets rich in fruit and vegetables have anti-inflammatory properties, which could protect lung function and may be an auxiliary tool in COVID-19 (Singh et al., 2022). However, whether diet is a pivotal factor related to the susceptibility and persistence of COVID-19 deserves further discussion. Regulating the gut microbiome by dietary interventions may act as a preventative and alternative strategy to minimize the severity and long-term implications of SARS-CoV-2. In this review, we will highlight the dynamic evolution of gut microbiota in acute COVID-19 to PACS, and further discuss the perspectives of diet as one of the possible prophylactic and therapeutic measures.

## 2 Perturbation of the gut-lung axis in COVID-19

The reciprocal communication between the gut and lung, which is mainly mediated by microbial metabolites and toxins translation through blood or lymphatic circulation, is known as the “gut-lung axis” (Synodinou et al., 2022). Gut microbiota dysbiosis may induce lung disorders and respiratory infections, by causing local or long-reaching immune, hormonal, and metabolic dysregulation (Dang and Marsland, 2019). Gut microbiota promotes granulocyte-macrophage colony-stimulating factor (GM-CSF) production, which activates extracellular signal-regulated kinase signaling in alveolar macrophages to enhance airway defenses (Brown et al., 2017). More specifically, the translocation of disordered microbial bacteria and derived metabolites may play a potential role in SARS-CoV-2 infection. The composition of gut microbiota was considered to be an important factor in regulating the expression of ACE2 in the gastrointestinal tract (Yang T. et al., 2020). Specific bacterial species, such as *Bacteroides dorei*, *Bacteroides thetaiotaomicron*, *Bacteroides massiliensis*, and *Bacteroides ovatus* (Geva-Zatorsky et al., 2017), were found to downregulate the expression of ACE2 and correlated inversely with fecal SARS-CoV-2 load (Zuo et al., 2020a). The intact intestinal epithelial cells could produce antiviral compounds that are hostile to viruses (Kalantar-Zadeh et al., 2020). The gut microbiome is also an important component of intestinal barrier function, which can defend against the invasion of potentially pathogenic bacteria (Bernard-Raichon et al., 2022). Epithelial barrier damage and gut microbiota disruption lead to gut pathogenic bacteria and their metabolites migrating to the lung (Chakradhar, 2017). COVID-19 patients were found to exhibit more occurrence of microbial translocation, especially for subjects admitted to the ICU (Oliva et al., 2021). Gut microbial dysbiosis could also influence the alteration of pulmonary flora during COVID-19. The translocation of microbiota and immune cells through the gut-lung axis may lead to more severe lung injury of COVID-19. Enrichment of gut bacteria in the lung microbiome was observed to be associated with sepsis and ARDS (Dickson et al., 2016).

The gut microbiome of patients with COVID-19 has impaired SCFA biosynthesis ability. SCFAs could move to the lung and promote the recruitment and maturation of immune cells locally (Anand and Mande, 2018). Treatment of gut epithelial organoids with butyrate could inhibit viral infection. A disintegrin and metalloproteinase 17 (ADAM17) serves as a metallopeptidase involved in ACE2 shedding and regulates its availability. Transmembrane protease serine 2 (TMPRSS2) facilitates virus-cell membrane fusion (Zlacká et al., 2021). SCFAs, especially butyrate, could downregulate the expression of the TMPRSS2 and ACE2 gene which is necessary for SARS-CoV-2 infection (Ohira et al., 2017), while upregulating the expression of ADAM17, thereby inhibiting viral infectivity to cells (Takabayashi et al., 2022). The kynurenine pathway of tryptophan metabolism was observed to activate in COVID-19 patients (Lionetto et al., 2021), which is involved in regulating inflammation and immune tolerance. Tryptophan metabolites can also cause the gut microbiota shift and indirectly regulate host intestinal inflammation and immune responses (Sonner et al., 2019). Furthermore, gut microbial metabolism of sphingolipid, lipopolysaccharide, and neutral amino acids was reported to increase in patients with COVID-19, which suggested the state of oxidative stress of gut microbiome in patients (Zhou et al., 2022). Thus, healthy gut microbiota and derived metabolites may enhance antiviral defenses, while inflammatory metabolism may exacerbate COVID-19.

**Figure 1:**
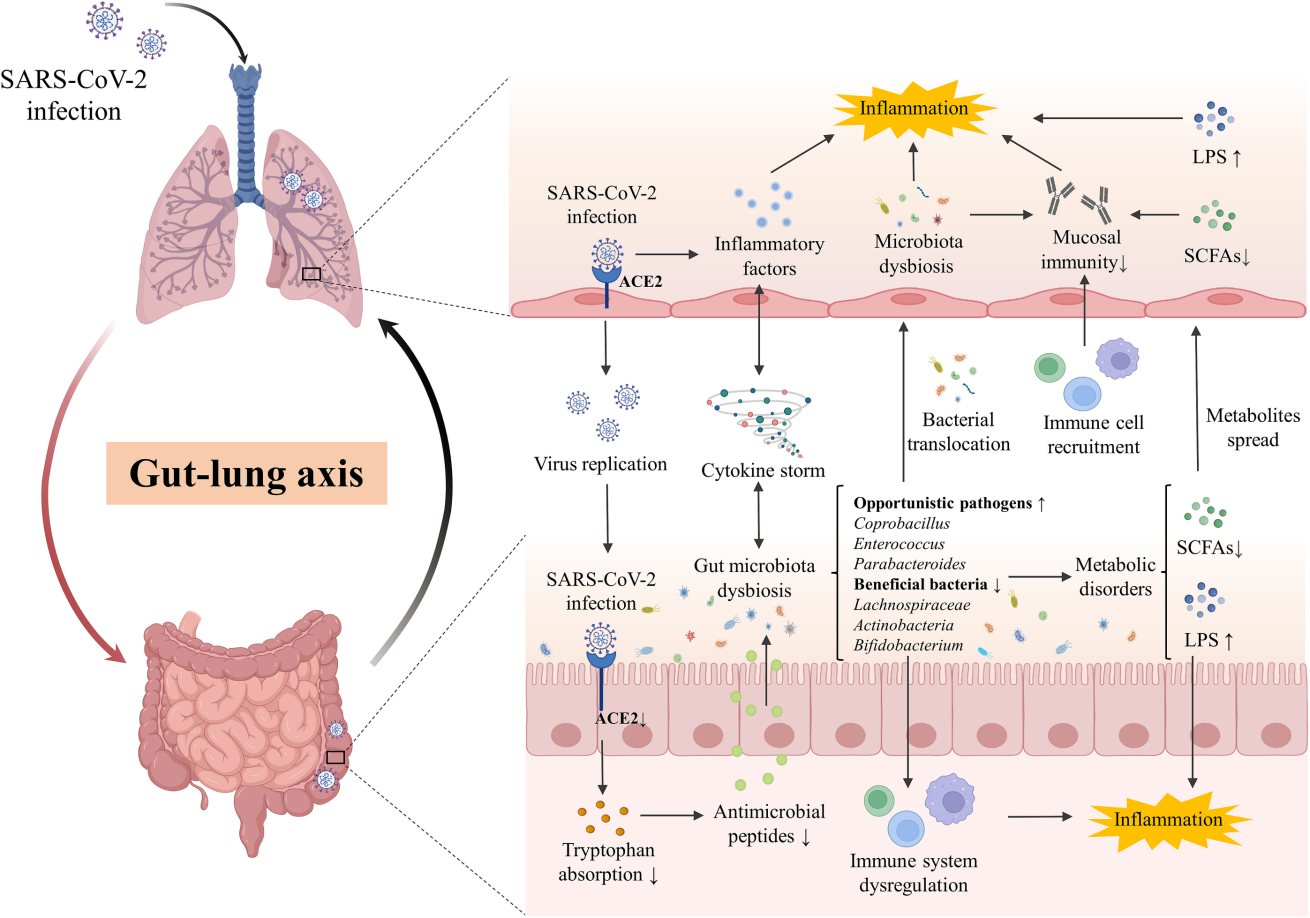
The gut-lung axis crosstalk in COVID-19. The key crosstalk between the gut microbiota and lung was termed the “gut-lung axis.” SARS-CoV-2 infected the lung by binding with ACE2 and downregulated the expression of intestinal ACE2, decreasing the transportation of tryptophan and the production of antimicrobial peptides, which then disturb the gut microbes and facilitate pathogen growth. Cytokine storm induced by SARS-CoV-2 infection in the lung could promote systematic or intestinal inflammation and contribute to gut microbiota dysbiosis. Opportunistic pathogens (eg., *Coprobacillus*, *Enterococcus*, *Parabacteroides*) were enriched while beneficial bacteria (eg., *Lachnospiraceae*, *Actinobacteria*, *Bifidobacterium*) were decreased in the gut of patients, inducing the disorder of derived metabolites including SCFAs and LPS. Disordered pathogens could translocate to the lungs to impact the local microecology and the recruitment of immune cells in the gut and lungs, affecting the local mucosal immune and inflammation. ACE2, angiotensin-converting-enzyme 2; LPS, lipopolysaccharide; SARS-CoV-2, severe acute respiratory syndrome coronavirus-2; SCFAs, short chain fatty acids.

Moreover, the increased severity of SARS-CoV-2 is associated with not only the virus but also an aggressive immune response. The concept of a common mucosal immunologic system has been proposed (McDermott and Bienenstock, 1979), and evidence is increasing in support of the hypothesis that microbiota may influence lung mucosal immune response by affecting intestinal mucosal immunity. Alteration of the gut microbiota or their metabolites can impair the immune activation against respiratory viruses including SARS-CoV-2. The damaged mucosa and shifted immune response cannot inhibit the overgrowth of pathogenic bacteria, further aggravating the pathophysiological state (Statovci et al., 2017). Interestingly, the interaction of the gut-lung axis is not restricted to a single way. The infection of the lungs can also indirectly increase the systemic pro-inflammatory factors and decrease oxygen levels, which induce intestinal inflammation, epithelial destruction, and antimicrobial peptide reduction, resulting in gut dysbiosis (Rastogi et al., 2022). The microbial dysbiosis of the respiratory tract could disrupt homeostasis and increase the load of gut microbiota due to bacterial translocation. Thus, the lung-gut axis is assumed as a bilateral loop, which would be regulated dynamically by the modulation of the lung or gut immune responses, and is perturbated in acute to post-acute COVID-19 syndrome (Figure 1).

### 2.1 Dynamics of gut microbiota in patients with acute to post-acute COVID-19 syndrome

SARS-CoV-2 infection has been attested to negatively affect the composition of gut microbiota, characterized by the increase of opportunistic pathogens and the reduction of beneficial commensals. The potential mechanisms of the gut microbiota alteration in COVID-19 have not been fully elucidated. Cytokine storm induced by pulmonary SARS-CoV-2 infection or intestinal impairment caused by direct gut infection could promote systematic or intestinal inflammation and contribute to gut microbiota dysbiosis (Merad and Martin, 2020; Guo Y. et al., 2021). Angiotensin-converting enzyme 2 (ACE2), which has been confirmed to have a high affinity with the coronavirus spike protein, is expressed not merely in the lung but also in the extrapulmonary organs including the gastrointestinal (GI) tract (Shang et al., 2020; Yan et al., 2020). SARS-CoV-2 infection could downregulate the expression of intestinal ACE2, which performed tryptophan transport and decreased the production of antimicrobial peptides, disturbing the ecology of gut microbes and then facilitating pathogen growth (Lecarpentier and Vallée, 2021; Penninger et al., 2021).

The sequencing studies required COVID-19 patients and healthy individuals to refrain from using antibiotics, probiotics, or a combination of both within 4 to 8 weeks before enrollment (Wu et al., 2021; Li S. et al., 2021; Yeoh et al., 2021), indicating that the gut microbiota exhibited significant differences in diversity and composition between patients with COVID-19 and healthy cohorts (Wu et al., 2021; Gu et al., 2020). Opportunistic pathogens were enriched and short-chain fatty acid (SCFA)-producing bacteria were exhausted in the patients, suggesting the universality of gut microbiota dysbiosis in COVID-19 infection (Yeoh et al., 2021). At the phylum level, *Bacteroides* was significantly increased and *Actinomycetes* was decreased in COVID-19 patients (Ren et al., 2021). *Faecalibacterium prausnitzii* (*F. prausnitzii*), which produced SCFAs and was positively correlated with a higher quantity of blood neutrophils, was reduced in patients with COVID-19 (Zhang F. et al., 2022). Furthermore, *Bacteroides stercoris*, *B. vulgatus*, *B. massiliensis*, *Streptococcus thermophilus* (*S. thermophilus*) were enriched in COVID-19 patients, while *Clostridium nexile*, *S. salivarius*, *Coprococcus catus*, *Eubacterium hallii*, and *Enterobacter aerogenes* were decreased. Four microorganisms, including *S. thermophilus*, *Bacteroides oleiciplenus*, *Fusobacterium ulcerans*, and *Prevotella bivia*, were identified only in patients with COVID-19 (Li S. et al., 2021). Even asymptomatic infants exhibited changes in gut microbiota, notably by decreasing anti-inflammatory bacteria including *Bifidobacterium bifidum* (*B. bifidum*) and *Akkermansia muciniphila* (Nashed et al., 2022). High fecal SARS-CoV-2 infectivity, which suggested active gastrointestinal SARS-CoV-2 infection, was associated with a higher abundance of *Collinsella aerofaciens*, *Collinsella tanakaei*, *Streptococcus infantis*, *Morganella morganii* in the gut (Zuo et al., 2021). Opportunistic fungal pathogens were found to increase in patients with COVID-19 as well, such as *Candida albicans*, *Candida auris*, and *Aspergillus flavus* (Zuo et al., 2020b) (Table 1).

**Table 1:** Gut microbiota alterations in patients with COVID-19.

ID	Country	Study characteristics/Sample type	Microbiome alterations
Wu et al. (2021)	China	140 throat swab samples and 81 stool samples from hospitalized COVID-19 patients, as well as 44 throat swab samples and 32 stool samples from healthy individuals	Comparison between COVID-19 and controls:Increased taxa: *Streptococcus*, *Weissella*, *Enterococcus*, *Rothia*, *Lactobacillus*, *Actinomyces*, *Granulicatella*, *C. citroniae*, *B. longum*, and *R. mucilaginosa* speciesDecreased taxa: *Blautia*, *Coprococcus*, *Collinsella*, *B. caccae*, *B. coprophilus*, *B. obeum,* and *C. colinum* species
Yeoh et al. (2021)	China	Two-hospital cohort study includes 10 COVID-19 patients and non-COVID-19 subjects	*Bacteroidetes* were more relatively abundant in COVID-19 patients, whereas *Actinobacteria* were more relatively abundant in non-COVID-19 individuals
Ren et al. (2021)	China	Cross-sectional study includes confirmed patients (24 fecal and 48 tongue-coating samples) and healthy controls (48 fecal and 100 tongue-coating samples)	Comparison between COVID-19 and controls:Increased taxa: *Streptococcus and Enterococcus genera*Decreased taxa: *Pseudobutyrivibrio*, *Ruminococcaceae uncultured*, *Blautia*, *Faecalobacterium*, *Bacteroides, Akkermansia*, *Lachnospiraceae incertae sedis,* and *Bifidobacterium taxa*
Li S. et al. (2021) and Li J. et al. (2021)	China	Cross-sectional study includes 47 fecal samples from COVID-19 patients and 19 healthy controls	Four specific microorganisms in COVID-19 patients: *Streptococcus thermophilus*, *Bacteroides oleiciplenus*, *Fusobacterium ulcerans,* and *Prevotella bivia*.Increased taxa: *B. vulgatus*, *Bifidobacterium longum*, *Streptococcus thermophilus*, *Prevotella bivia, Erysipelotrichaceae bacterium* 6,145, and *Erysipelotrichaceae bacterium* 2244ADecreased taxa: *Streptococcus salivarius*, *Coprococcus catus*, *Eubacterium hallii*, *Enterobacter aerogenes,* and *Adlercreutzia equolifaciens*
Moreira-Rosário et al. (2021)	Portugal	A multicenter cross-sectional prospectively study includes 115 COVID-19 patients, classified as: mild patients 19, moderate patients 37, and severe patients 59	Gut microbiota of moderate and severe patients is characterized by lower *Firmicutes/Bacteroidetes* ratio, higher abundance of *Proteobacteria* and lower abundance of beneficial butyrate-producing bacteria such as the genera *Roseburia* and *Lachnospira*
Zuo et al. (2020a,b)	China	A prospective study, 15 COVID-19 patients, 6 pneumonia patients, and 15 healthy individuals	Increased taxa: *Clostridium hathewayi*, *Actinomyces viscosus,* and *Bacteroids nordii*Decreased taxa: *Eubacterium ventriosum*, *Faecalibacterium prausnitzii*, *Roseburia,* and *Lachnospiraceae taxa*
Zhang et al. (2023)	China	A two-sample Mendelian randomization analysis included 18,340 participants from 24 cohorts	*Ruminococcaceae UCG013* genus and *Ruminococcus1* genus were suggestively associated with COVID-19Severe COVID-19 was significantly related to *Turicibacter* and *Olsenella* genus, and potentially associated with *Ruminococcus1*, *CandidatusSoleaferrea*, and *Parasutterella* genus

Surprisingly, the gut microbiome characteristics of COVID-19 were largely restored following the disease subsided but did not fully revert to normal. The microbial diversity gradually increased in the recovery process especially butyric acid-producing microbes and *Bifidobacterium* while LPS-producing microbes decreased (Cui et al., 2022). *F. prausnitzii*, *Eubacterium rectale* (*E. rectale*), and *Bifidobacteria* are less abundant in patients 1 month after the infection is resolved. However, patients with PACS still had significant microbial alterations at 6 months after acute infection, which was characterized by a higher abundance of *Ruminococcus gnavus* (*R. gnavus*), *Bacteroides vulgatus* and lower abundance of *F. prausnitzii* (Liu et al., 2022a). The alterations of gut microbiome linger beyond 1 year, showing enrichment of potentially pathogenic bacteria such as *Erysipelatoclostridium ramosum* and *R. gnavus*, while a depletion of beneficial bacteria such as *B. adolescentis* and *B. pseudocatenulatum* (Su et al., 2023). *Enterobacteriaceae* strains with antibiotic-resistance were found to dominate in the gut of post-COVID patients along with the reduced SCFAs in feces. Fecal microbiota transplantation (FMT) from post-COVID subjects to germ-free mice resulted in exacerbated lung inflammation and brain dysfunction. *A. flavus* and *Aspergillus niger*, as respiratory fungal pathogens, were still detected in the feces of COVID-19 patients after SARS-CoV-2 clearance and respiratory symptoms resolution (Zuo et al., 2020b). Therefore, gut microbiota may directly contribute to the prolonged sequelae of COVID-19, suggesting its potential as a therapeutic target (Mendes de Almeida et al., 2023) (Table 2).

**Table 2:** Gut microbiota alterations in patients with post-acute COVID-19 syndrome.

ID	Country	Study characteristics/Sample type	Microbiome alterations
Liu et al. (2022a,b)	China	A prospective study 106 patients with COVID-19 severity from hospital admission to 6 months, along with 68 non-COVID-19 controls	PACS patients had higher levels of *Ruminococcus gnavus*, *Bacteroides vulgatus* and lower levels of *Faecalibacterium prausnitzii*Butyrate-producing bacteria, including *Bifidobacterium pseudocatenulatum* and *Faecalibacterium prausnitzii* showed negative correlations with PACS at 6 months
Su et al. (2023)	China	A prospective study involving 155 patients with COVID-19 and 155 non-COVID-19 patients	Enriched microbiota in PACS: *R. gnavus, C. bolteae,* and *E. ramosum*Decreased microbiota in PACS: *G. formicilis* and *B. adolescentis*
Mendes de Almeida et al. (2023)	Brazil	Cross-sectional study of 72 post-COVID subjects and 59 healthy controls	Compared to controls, post-COVID subjects had a higher percentage of *Enterobacteriaceae* strains with drug-resistant and multidrug-resistant phenotypes
Liu et al. (2022a,b)	China	A prospective follow-up study over 6 months of 296 fecal metagenomes and 79 fecal metabolomes	PACS has an increase in the diversity of opportunistic pathogenic bacteria, including *Erysipelatoclostridium ramosum*, *Clostridium bolteae,* and *Clostridium innocuum* at 6 months
Vestad et al. (2022)	Norway	149 COVID-19 participants attended the follow-up for 3 months after admission	Patients with respiratory dysfunction had reduced abundance of *Erysipelotrichaceae UCG-003* and increased abundance of *Veillonella* and *Flavonifractor*

### 2.2 Gut microbiota was associated with the severity and prognosis of COVID-19

Based on millions of confirmed cases worldwide, several risk factors have been identified to be associated with the disease severity of COVID-19, including older age, male sex, ethnic variations, and underlying comorbidities. In addition, laboratory indices, pro-inflammatory cytokine levels, and complications are also the predictive factors for the progression of COVID-19 into a severe and critical stage. In contrast, a healthy diet and sufficient nutrition, COVID-19 vaccination, and atopic conditions may avoid the disease progression and poor outcome (Zhang et al., 2023). However, numerous studies have found that gut microbiota dysbiosis could negatively impact the recruitment of immune cells and the development of immune responses in the lungs, which could contribute to respiratory tract infections (Baradaran Ghavami et al., 2021). It has been demonstrated that COVID-19 severity is indeed highly correlated with the characteristics of gut microbiota, which can be utilized as a predictive biomarker and provide potential treatment strategies (Cao et al., 2021). Even after the SARS-CoV-2 clearance, persistent disturbance of the gut microbiota may be a pivotal factor for protracted symptoms and multisystem inflammation syndromes (Yeoh et al., 2021).

The gut microbiome in SARS-CoV-2 infected patients was associated with a pro-inflammatory signature, and the connectivity of an anti-inflammatory bacterial network was observed to reduce in patients with severe COVID-19 (Reinold et al., 2021). A multicenter cross-sectional study has shown that the gut microbiota of moderate and severe COVID-19 patients tended to have decreased *Firmicutes*/*Bacteroidetes* ratio, higher abundance in *Proteobacteria*, lower abundance in butyrate-producing bacteria from the family *Lachnospiraceae* and the phylum *Actinobacteria* (Moreira-Rosário et al., 2021). In the gut, significantly fewer *Bacteroides* and increased *Enterococcus* are found in intensive care unit (ICU) patients (Shen et al., 2022). The baseline abundance of *Coprobacillus*, *Clostridium ramosum*, and *Clostridium hathewayi* was positively associated with the severity of COVID-19 while the abundance of *F. prausnitzii* and *Alistipes onderdonkii* showed an inverse correlation with the disease severity (Zuo et al., 2020a). Severe COVID-19 was significantly associated with the *Turicibacter* and *Olsenella* genera and potentially linked to *Ruminococcus1*, *CandidatusSoleaferrea*, and *Parasutterella* genus (Zhang and Zhou, 2023). Two bacterial species *Oribacterium* sp. GMB0313 and *Ruminococcus* sp. GMB0270 are both observed to associated with COVID-19 resistance, and the pair of which protect against SARS-CoV-2 infection by activating CD8^+^ T cell-mediated immunity (Wang M. et al., 2024). Surprisingly, gastrointestinal fungal dysbiosis is also present in COVID-19 patients. The overgrowth of intestinal *Candida* and the elevated levels of *Candida albicans* IgG antibodies symbolized the severity of the disease. The transcriptional level of antifungal immunity pathways and reprogramming of granulocyte myeloid progenitors were increased, suggesting that gut fungal pathobionts may activate the immune response during COVID-19 (Kusakabe et al., 2023).

Poor basic physical status and severe acute COVID-19 convey the increased risk for the development of post-acute COVID-19, but mild COVID-19 may also culminate in post-acute sequelae (Xie et al., 2021). Species of the gut microbiome associated with host immune response modulation, such as *F. prausnitzii*, *E. rectale,* and *Bifidobacterial*, were decreased in patients even in post-COVID. More significantly, these perturbed compositions represented stratification with disease severity, plasma concentrations of inflammatory factors, and blood markers of tissue damage (Yeoh et al., 2021). Furthermore, the composition of gut microbiota was also observed to be associated with complications and mortality in COVID-19 patients. The gut bacterial richness was decreased with the number of complications. *Parabacteroides* was increased in patients with ARDS and hemodialysis, which suggested a more complicated course and a positive correlation to mortality. On the contrary, butyrate-producing bacteria, such as *F. prausnitzii*, were significantly reduced in patients with multiple complications (Liu et al., 2022b; Schult et al., 2022). An observational prospective cohort study had shown that the increase of *Enterococcus* spp., *Staphylococcus aureus* and *Candida* spp. both in oropharyngeal and rectal samples were associated with a 17% or greater higher risk of death (Patrier et al., 2022). Mortality from COVID-19 was demonstrated to be associated with enriched *Proteobacteria* in the feces and decreased secondary bile acids (Stutz et al., 2022). Thus, a balanced and robust gut microbiome may propel a favorable outcome for COVID-19 individuals. The gut bacterial signatures can be used for disease diagnosis and mortality estimation.

### 2.3 The ongoing disruption of gut-lung crosstalk in post-acute COVID-19 syndrome

**Figure 2:**
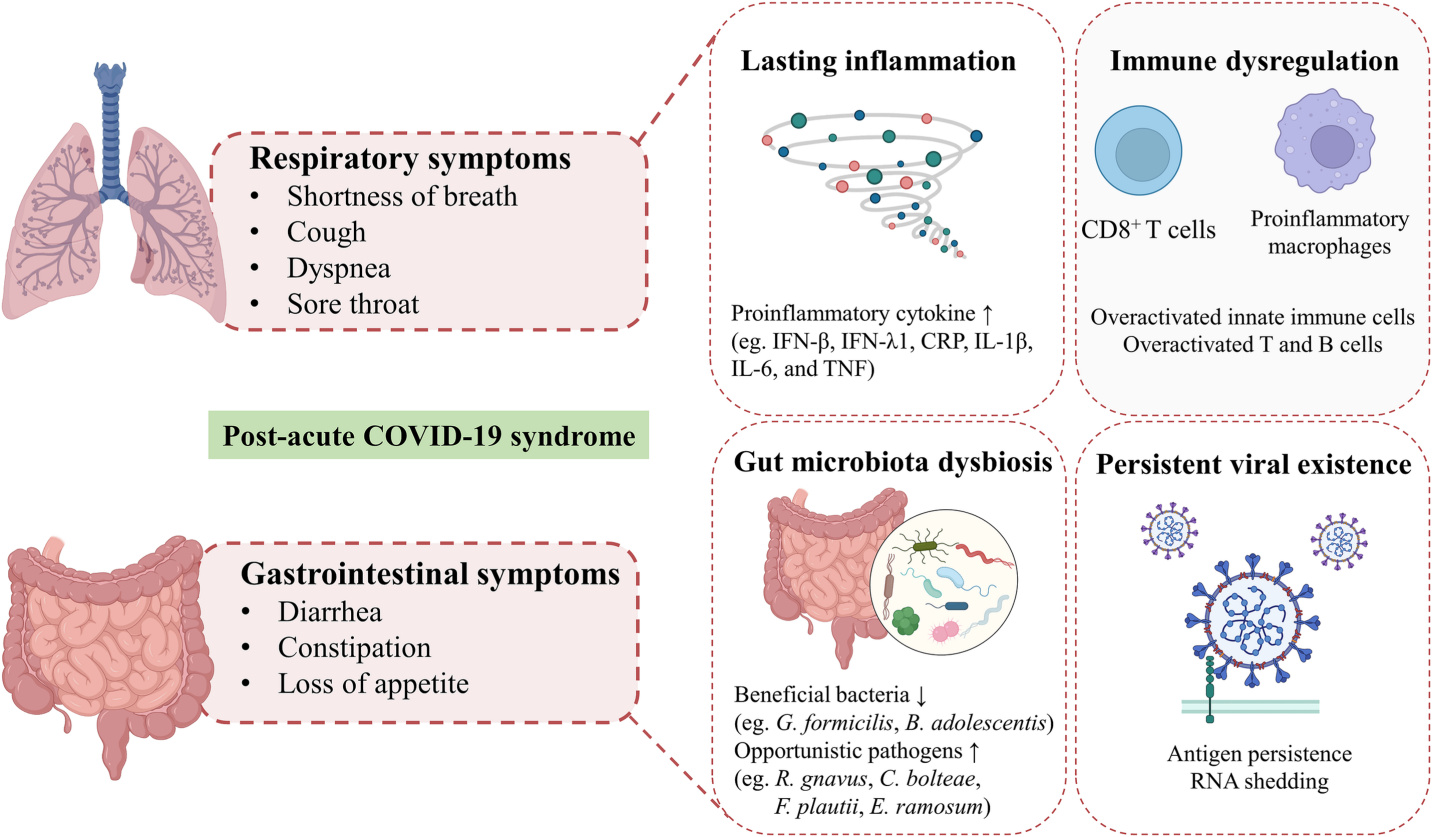
Hypothesized mechanisms of respiratory and gastrointestinal post-acute COVID-19 syndrome. Shortness of breath, cough, dyspnea and sore throat are the common respiratory symptoms in patients with post-acute COVID-19 syndrome. GI symptoms of PACS, including diarrhea, constipation, loss of appetite are mainly presented. The exact mechanisms of PACS may be multiply overlapping, several hypothesized mechanisms mainly including persistent viral existence in tissues, gut microbiota alteration, long-lasting inflammation and immune dysregulation.

Individuals with PACS may experience over 50 persistent symptoms, including fatigue, dyspnea, dry coughing, olfactory and gustatory dysfunctions, diarrhea, nausea, and abdominal pain, which may fluctuate or relapse over time. The exact mechanisms of PACS may be multiply overlapping, several hypotheses have been proposed, including persistent viral existence in tissues, dysfunction of vascular endothelium and nervous system, reactivation of latent pathogens and gut microbiota alteration, long-lasting inflammation, and immune dysregulation (Davis et al., 2023). The high abundance of ACE2 receptors on intestinal cells supports the persistent viral reservation, which may be a potential mechanism of PACS (Lee et al., 2020). Intestinal affinity to SARS-CoV-2 was correlated with gastrointestinal symptoms in the early stage and may increase the risk of developing GI disorders even after COVID-19. More importantly, disruptions in the intestinal barrier and imbalances in gut microbiota could further result in an increase of bacterial lipopolysaccharide and peptidoglycan at the systemic level to amplify systemic inflammation among patients with PACS persistently (Teixeira et al., 2021; Schultheiß et al., 2022). The toxins generated by leaky gut microbiota and uraemic solutes generated by the kidney with chronic inflammation, potentially lead to symptoms such as fatigue, disturbances in mineral bone metabolism, neurological issues, and compromised cardiovascular function (Adhikari et al., 2024). Recent studies have revealed that gut microbiota alterations, mucosal inflammation, and intestinal permeability were associated with metabolic disorders in PACS. SARS-CoV-2 can trigger specific metabolic conditions and can push a compromised system into a positive feedback loop, contributing to limited mitochondrial dysfunction, including down-regulation of core mitochondrial genes and unusual swelling of cristae (Guarnieri et al., 2023; Cortese et al., 2020). Mitochondrial dysfunction impedes the process of antioxidants and respiratory chain, which then affect the oxidative phosphorylation and cellular signaling cascades (Mandò et al., 2021). Similar mitochondrial dysfunction has been observed in myalgic encephalomyelitis/chronic fatigue syndrome (ME/CFS) of PACS, which may result from the reactivation of EBV (Vernon et al., 2006). However, the heterogeneity and complexity of PACS hindered the decryption of the pathophysiological mechanisms, which need further multidisciplinary studies to better understand and address these underlying health problems. The involvement of coexisting symptoms suggests the requirement to provide insights into different symptom clusters and identify potential targets for personalized therapeutic interventions. In this review, we highlighted the persistent respiratory and gastrointestinal symptoms in PACS and discussed underlying mechanisms involved in the gut-lung crosstalk (Figure 2).

Shortness of breath and cough are the most common respiratory symptoms in patients with long-COVID at 7 months (Davis et al., 2021), and 43.4% of patients showed persisting dyspnea at 60 days (Carfì et al., 2020). Severe COVID-19 patients often present long-term pulmonary sequelae, which are accompanied by decreased lung function, systemic inflammation, and persistent respiratory immune imbalance (Littlefield et al., 2022). Specifically, dysregulated responses of potential pathogenic subsets of CD8^+^ T cells were associated with impaired lung function after acute COVID-19 (Cheon et al., 2021). SARS-CoV-2 specific CD8^+^ T cells in the nasal mucosa were found to persist 2 months after viral clearance, indicating the long-term effects of COVID-19 on immune reactions in the upper respiratory tract (Roukens et al., 2022). Respiratory dysfunction at 3 months after COVID-19 was related to the altered composition of gut microbiota, such as reduced *Erysipelotrichaceae* UCG-003 and increased *Veillonella* and *Flavonifractor*, which persistently elevated the plasma levels of gut barrier dysfunction marker lipopolysaccharide-binding protein (LBP) (Vestad et al., 2022). Thus, the gut-lung axis may be involved in the development of respiratory symptoms of PACS persistently.

Along with respiratory injury, about 15% of patients presented GI manifestations during acute infection, including nausea or vomiting, diarrhea, and loss of appetite (D'Amico et al., 2020; Mao et al., 2020), even as the onset symptom (Song et al., 2020). The feces of COVID-19 patients were detected to have more nutrients that should be metabolized or absorbed and harmful metabolites. Fecal sucrose content is increased and glucose content is decreased in patients with COVID-19 due to sucrase-isomaltase insufficiency, which may cause typically manifest, such as osmotic diarrhea with vomiting, flatulence, and abdominal pain (Lv et al., 2021). In patients with PACS, GI symptoms are increasingly recognized as an important public health concern (Meringer and Mehandru, 2022). Functional dyspepsia-like and irritable bowel syndrome (IBS)-like post-COVID GI symptoms were found in approximately 40% of patients (Vélez et al., 2022), which may be associated with the increased permeability of gut cells caused by gut microbiome dysbiosis, adverse effects of antiviral drugs, and inflammatory response to the virus. The altered gut microbiome activated the immune response and affected neuromuscular function, causing IBS-like symptoms in GI PACS (Freedberg and Chang, 2022). Anxiety after COVID-19 frequently occurred and was associated with an increased risk of GI symptoms, especially for patients with mental health symptoms before COVID-19 (Blackett et al., 2022). SARS-CoV-2 antigen persistence in the gut after resolution may play a potential role in this process. SARS-CoV-2 RNA was detected in the gut mucosa and viral nucleocapsid protein persisted in gut epithelium and CD8^+^ T cells after mild acute COVID-19 infection in inflammatory bowel diseases (IBD) patients. The majority of patients with persistent viral antigens reported sequelae of COVID-19, which indicated viral antigen persistence may be the basics of PACS (Zollner et al., 2022). Intestinal biopsies obtained from patients at 4 months after acute COVID-19 still revealed the persistence of SARS-CoV-2 nucleic acids and immunoreactivity (Gaebler et al., 2021). However, Park et al. (2021). demonstrated that SARS-CoV-2 RNA shedding in the feces of patients at 2 months was common but had no association with GI symptoms, and the virus was detected to clear from the gut by 6 months. Therefore, whether GI PACS is driven by sustained viral replication in the gut directly remains skeptical (Freedberg and Chang, 2022).

Almost all PACS were associated with the depletion of beneficial bacteria in gut microbiota such as *Gemmiger formicilis* and *B. adolescentis*, while the enrichment of potential pathogens such as *R. gnavus*, *Clostridium bolteae*, *Flavonifractor plautii* and *E. ramosum* were associated with the majority of the PACS. Depletion of several profitable bacteria, such as *B. adolescentis*, was associated with specific symptoms post-acute COVID-19 (Su et al., 2023). The gut microbiome was found to be associated with the phenotypic manifestations of PACS, which suggested the potential clinical utility for its prediction and diagnosis (Su et al., 2024). Moreover, fungal translocation, which was measured as *β*-glucan, was elevated in the plasma of long-COVID patients, possibly inducing higher NF-κB signaling and cytokine production, indicating a potential target linking fungal translocation and inflammation in PACS (Giron et al., 2022). Gut microbiota dysbiosis modifies the immune response and may affect the recovery from PACS among multiple systems far beyond the GI tract.

Ulteriorly, the mechanisms of PACS involve viral infection-induced inflammatory and immune responses (Ruf, 2024). Nucleocapsid IgG levels at 3 months post-infection and neutralizing capacity at 8 months were detected to elevate in long-COVID patients. Spike-specific and nucleocapsid-specific CD4^+^ T cells as well as TIM-3 expression on CD4^+^ and CD8^+^ T cells were observed to increase at 3 and 8 months, but return to comparable immune responses over 24 months (Phetsouphanh et al., 2024). Post-acute COVID-19 was related to hyperactivated innate immune cells and T and B cells, along with elevated expression of the proinflammatory cytokine, such as IFN-β and IFN-λ1 that remained persistently high at 8 months after infection (Phetsouphanh et al., 2022). The IL-1β, IL-6, and TNF cytokine triad was found to be associated with PACS and create self-sustaining feedback in pro-inflammatory macrophages (Schultheiß et al., 2022). C-reactive protein (CRP) levels were detected to increase persistently during SARS-CoV-2 infection to PACS, indicating the long-term symptoms may be related to a specific increase in the CD8^+^ T cell response. The peripheral immune system of COVID-19 convalescents has a subset of changes for at least 6 months post-infection, which were associated with long-COVID (Ryan et al., 2022). Notably, SARS-CoV-2-specific T cells are differently activated in distinct PACS. SARS-CoV-2 specific CD8^+^ T cells exhibited cytotoxic characteristics for GI PACS, while T cells followed the opposite trend in patients with respiratory symptoms. The cytotoxic T cells and newly emerging cytotoxic CD4^+^ T cells were enriched in patients with GI PACS, indicating its correlation with unique T cell clonal and transcriptome (Su et al., 2022). Specific inflammatory pathways and markers were observed to be implicated in subtypes of long-COVID, such as IL-1R2, MATN2 and COLEC12 were associated with cardiorespiratory symptoms, fatigue and anxiety (Liew et al., 2024). These findings indicate that managing long-COVID patients on account of the different subphenotypes may be more effective and support the use of immunomodulatory agents.

## 3 Dietary patterns modulate the gut microbiome and impact acute to post-acute COVID-19 syndrome

**Figure 3:**
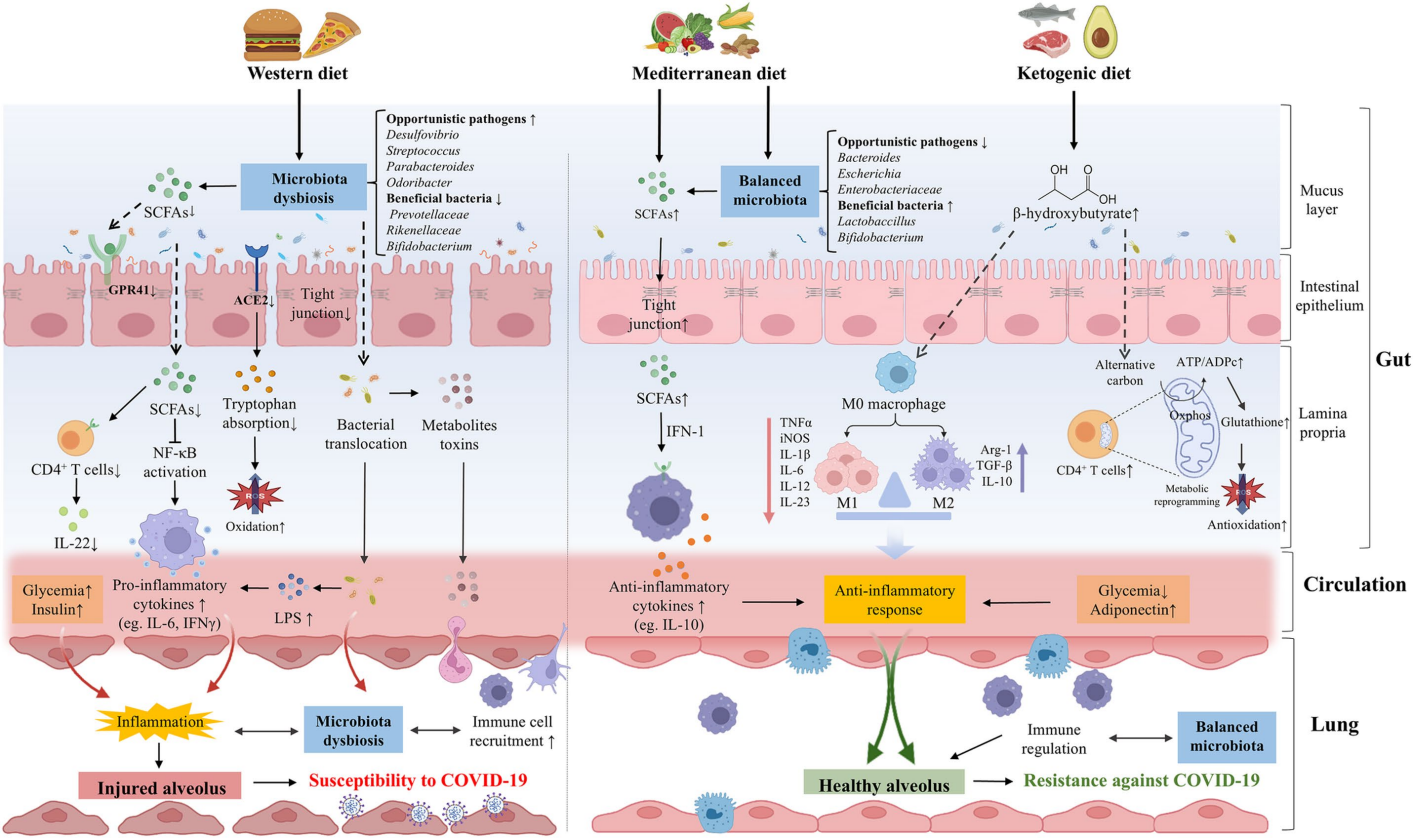
The effects of dietary patterns on the pathogenesis or prevention of COVID-19. WD intake significantly altered the gut microbial composition, opportunistic pathogens (eg., *Desulfovibrio*, *Streptococcus*, and *Parabacteroides*, *Odoribacter*) were increased while beneficial bacteria (eg., *Prevotellaceae*, *Rikenellaceae*, and *Bifidobacterium*) were decreased. WD could destroy the intestinal barrier integrity and promote the bacteria invasion and excessive production of metabolite toxins. The composition of gut microbiota was important in downregulating the expression of ACE2, reducing the absorption of tryptophan and increasing oxidative stress. Meanwhile, WD could reduce the abundance of SCFAs-producing bacteria and the production of SCFAs, thereby facilitating the secretion of pro-inflammatory cytokines and dysregulating immune function, which can increase the susceptibility of lungs to COVID-19 in various ways. A healthy diet such as MD and KD may be beneficial to COVID-19. MD balanced the gut microbiota, including the increase of beneficial bacteria (eg., *Lactobaccillus* and *Bifidobacterium*) and the decrease of opportunistic pathogens (eg., *Bacteroides*, *Escherichia,* and *Bifidobacterium*). MD can be metabolized by gut microbiota to produce SCFAs, which mediate systematic immunoregulation and anti-inflammatory response through IFN-I signaling. Ketone bodies such as BHB can balance the structure of the intestinal flora and restore the intestinal barrier. BHB not only induces M2 macrophage polarization to exert anti-inflammatory effects, but also promotes oxidative phosphorylation, reshaping the mitochondrial reoxidation-reduction balance and restoring the immune function of CD4^+^ T cells, thereby enhancing resistance against SARS-CoV-2 infection. WD could directly contribute to hyperglycemia and hyperinsulinemia, promoting the formation of lung inflammation. On the contrary, healthy diets are linked to lower glycemia and higher concentrations of adiponectin, which have an anti-inflammatory effect. ACE2, angiotensin-converting-enzyme 2; BHB, *β*-hydroxybutyrate; COVID-19, coronavirus disease 2019; IFN-1, interferon-1; KD, ketogenic diet; LPS, lipopolysaccharide; MD, Mediterranean diet; SARS-CoV-2, severe acute respiratory syndrome coronavirus-2; SCFAs, short chain fatty acids; WD, Western diet.

The composition of gut microbiota could be dynamically altered by numerous factors, including diet, lifestyle, disease, and aging (Zuo et al., 2020a; Zmora et al., 2019). It is well-established that diet impacts the gut microbiota structure and function rapidly but persistently. Short-term consumption of an animal-based diet consisting of meat, eggs, and cheese increased the abundance of bile-tolerant microorganisms (*Alistipes*, *Bilophila*, and *Bacteroides*) and reduced the levels of Firmicutes (*Roseburia*, *E. rectale*, and *Ruminococcus bromii*) that metabolize dietary plant polysaccharides (David et al., 2014). Although there is no specific dietary supplementation that has been shown to have exact benefits for COVID-19 treatment or prevention, dietary patterns have been shown to be associated with the infection risk, severity, and even consequences (Merino et al., 2021; Yue et al., 2022; Bell et al., 2023). Although the recovery of PACS is frequently complicated by sustained dysfunction, such as fatigue, dysphagia, appetite loss, and taste alterations, making the appropriate evaluation of dietary intake difficult management for these patients, personalized dietary recommendations represent one of the optimal strategies for PACS recovery (Barrea et al., 2022). Thus, encompassing the role of diet in gut microbiota modulation and being mindful of dietary patterns may reduce susceptibility to and long-term complications from COVID-19 (Butler and Barrientos, 2020) (Figure 3).

### 3.1 Western diet

The Western diet (WD) is high in saturated fats, sugars and refined carbohydrates, which can lead to gut dysbiosis and profound implications (Reimer, 2019; Liu et al., 2020a). High-fat diet (HFD) increased the *Firmicutes*/*Bacteroidetes* ratio and opportunistic pathogens including *Desulfovibrio*, *Streptococcus*, *Parabacteroides,* and *Odoribacter*, while decreasing the beneficial bacteria, such as *Prevotellaceae*, *Rikenellaceae* and *Bifidobacterium*, which were negatively correlated with gut barrier function (Liu et al., 2020b; Kociszewska et al., 2021). HFD was demonstrated to reduce the abundance of *Roseburia* and *Lachnospiraceae bacterium* (Liu et al., 2020b), which were significantly altered in the fecal microbiome of COVID-19 patients (Zuo et al., 2020a). Furthermore, HFD-induced microbiota dysbiosis disrupted the intestinal barrier, increasing gut pathogenic bacteria and circulating endotoxin, which disequilibrated the local microenvironment and caused inflammation in the lung (Chakradhar, 2017). COVID-19 patients were found to exhibit more occurrence of microbial translocation, especially for subjects admitted to the ICU (Oliva et al., 2021). Diet intake or gut microbial dysbiosis could also influence the alteration of pulmonary flora. The translocation of microbiota and immune cells through the gut-lung axis may lead to more severe lung injury of COVID-19 (Kruglikov et al., 2020). Enrichment of gut bacteria in the lung microbiome was observed to be associated with sepsis and ARDS (Dickson et al., 2016). In addition to the effects of bacteria, the metabolites of gut microbiota can also transfer and activate the immune response. LPS produced by most Gram-negative bacteria could activate the NF-κB pathway and induce severe lung injury, which is a potentially critical determinant of the pathobiology in ARDS (Everhart et al., 2006). HFD could also promote the overgrowth of pathogenic bacteria in the intestine and induce the maturation of proinflammatory immune cells, leading to uncontrolled inflammation and mucosal damage indirectly, which were unable to inhibit the overgrowth of pathogenic bacteria, further aggravating the pathophysiological state (Statovci et al., 2017). Gut dysbiosis could impact the recruitment of immune cells in the lungs through the gut-lung axis. Mucosa-associated invariant T (MAIT) cells are antimicrobial T cells to recognize bacterial metabolites and play a part in antiviral responses, which were activated in the circulation and enriched in the airways of patients with COVID-19 (Parrot et al., 2020).

Long-term WD composition impaired insulin clearance through a small cluster of gut microbes or their metabolites during the progression of obesity and diabetes (Foley et al., 2020), which could place these populations at an increased risk for severe COVID-19 and PACS (Turnbaugh, 2017; Flint and Tahrani, 2020; Wang C. et al., 2023). Both obesity and diabetes can lead to lung function impairment by causing chronic inflammation and delaying ineffective immune response. The risk of hospitalization and death due to COVID-19 showed a linear increase at BMI above 23 kg/m^2^ and the risk of ICU admission increased linearly across the whole BMI range (Gao et al., 2021). Adipose tissue may be a major player in the spread of SARS-CoV-2 and systemic immune activation. The overexpression of inflammatory adipokines can impair chemotaxis, alter macrophage differentiation, and upregulate inflammatory cytokines such as IL-6 to contribute to the increased morbidity of COVID-19 (Dietz and Santos-Burgoa, 2020; Malavazos et al., 2020). Diet-induced obesity and non-alcoholic steatohepatitis (NASH) were found to impair the recovery from COVID-19 (Briand et al., 2022). Furthermore, the vaccine for SARS-CoV-2 was found to be less effective in obese individuals compared with healthy-weight individuals (Ealey et al., 2021). Remarkably, diabetes was found to be positively associated with COVID-19 severity and complications incidence post-COVID, which may be attributed to systemic inflammation, metabolite disorders (Heintz-Buschart et al., 2016), and the effects of innate immune deficiency (Bornstein et al., 2020). On the contrary, participants missing PACS were more likely to have lower BMI and be less likely to have type 2 diabetes, which may be related to less release of cytokines after infection and long-term complications of multiple organs subsequently (Wang S. et al., 2023).

Severe patients with COVID-19 more frequently had increased inflammatory monocytes and neutrophils and a sharp decrease in lymphocytes (Giamarellos-Bourboulis et al., 2020), with higher levels of IL-1β, IL-6, and TNF-*α* (Chen G. et al., 2020), leading to systemic organ failure and tissue damage. Diet may have a widespread role in regulating the immune response to SARS-CoV-2 infection and consequently modulate the disease severity (Morais et al., 2021). WD consumption could activate innate and inhibit adaptive immunity, inducing chronic inflammation and impairing the defense against viruses. HFD was observed to increase macrophage infiltration in alveoli, which is strongly associated with ARDS and poor outcomes in COVID-19 patients (Purbey et al., 2023). For the adaptive immune system, WD consumption induced oxidative stress to consume the number and inhibit the function of T and B lymphocytes, which led to immunodepression and was associated with more severe pathogenesis of COVID-19 (Butler and Barrientos, 2020). Excessive production of proinflammatory molecules, which is called the cytokine storm, may be aggravated by WD with high pro-inflammatory potential. Severe COVID-19 was found to have higher concentrations of inflammatory markers than mild patients (Ji et al., 2020). The pro-inflammatory blood markers were observed to be higher and the CD8^+^ T cell number was lower in patients with severe COVID-19 illness (Reinold et al., 2021). Moreover, peripheral inflammation post-COVID may have long-term consequences, such as dementia and neurodegenerative disease, which can be further promoted by an unhealthy diet (Butler and Barrientos, 2020).

### 3.2 Mediterranean diet

On the contrary, the Mediterranean diet (MD) is rich in fiber, antioxidants, vegetable protein, vitamins, and minerals. A statistical analysis included a cohort of 5,194 participants and found that higher adherence to the MD may be associated with a lower subsequent risk of COVID-19 (Schwingshackl and Hoffmann, 2014). The increased MD score values reduced the rate of COVID-19 occurrence and were observed to have a negative association with both COVID-19 cases and related deaths (El Khoury and Julien, 2021; Greene et al., 2021). The severity of SARS-CoV-2 infection was associated with higher age and saturated fat intake, while lower MD and cereal consumption (Ponzo et al., 2021). A comparative study among vegetarians, vegans and omnivorous diet subjects showed that the abundance of *Bacteroides*, *Escherichia coli* and *Enterobacteriaceae* in vegetarians was significantly reduced compared with the omnivorous control group, while the subjects on vegetarians ranked between vegans and controls (Zimmer et al., 2012). Dietary fiber has been reported to increase the diversity of gut microbiota related to suppressive mucosal inflammation and promoted health, such as *Bifidobacterium* and *Lactobaccillus* (Carlson et al., 2018). *Firmicutes* and *Proteobacteria* were higher while *Bacteroidetes* was lower in mice fed by fiber deficiency diet, which eroded the colonic mucus barrier, decreased the level of SCFAs, and disrupted the mucosal barrier integrity (Neumann et al., 2021). Dietary fibers could be fermented by some species of gut microbiota and produce SCFAs, which provide energy to intestinal epithelium cells and strengthen the intestinal barrier, preventing inflammation induced by LPS (Seethaler et al., 2022). Increased concentrations of SCFAs in systemic circulation play an anti-inflammatory role far beyond the gut by immune modulation (Yao et al., 2022). Cao et al. (2021) found that severe COVID-19 cases showed depletion of butyrate-producing bacteria compared with mild to moderate cases. Butyrate increases the expression of TLRs which are involved in antiviral mechanisms and may also inhibit SARS-CoV-2 replication by downregulating high-mobility group box protein-1 (HMGB1) expression (Li J. et al., 2021). Dietary fiber intake is negatively correlated with the serum levels of inflammatory markers, including CRP, IL-6 and TNF*α*, which are massively released in COVID-19. Furthermore, MD can influence sex-specific responses to and the tiredness side effect of the SARS-CoV-2 vaccine, which suggests the significance of individual dietary recommendations on enhancing vaccine responses (Gualtieri et al., 2024).

Dietary recommendations for PACS include several foods with anti-inflammatory and immuno-stimulating activities, and MD might be a valuable strategy to achieve (Barrea et al., 2022). Patients were found to better recover from COVID-19 with a diet enriched in fiber, which is mediated by SCFAs in systematic immunoregulation and anti-inflammatory response through IFN-I signaling. Long-term sequelae of COVID-19 subjects had a remarkable predominance of *Enterobacteriaceae* strains in the gut with reduced SCFA levels in feces (Mendes de Almeida et al., 2023). These disorders persist even after disease recovery, which positively correlate with disease severity and increased plasma concentrations of CXCL-10, NT-proB-type natriuretic peptide, and CRP (Zhang F. et al., 2022). In addition, SCFAs could move to the lung and promote the recruitment and maturation of immune cells locally (Anand and Mande, 2018). Specifically, SCFAs exert immunomodulatory effects by engaging with receptors on immune cells, such as GPR41 and GPR43, inhibiting the generation of pro-inflammatory cytokines while enhancing anti-inflammatory cytokines like IL-10 and antioxidant enzymes (Sungnak et al., 2020; Yang W. et al., 2020). Furthermore, high-fiber diets are linked to lower glycemia and higher concentrations of adiponectin, which have an anti-inflammatory effect. Inhibition of dipeptidyl peptidase 4 (DPP4) could suppress T-cell proliferation and IL-6 and IL-10 secretion, which regulate the immune response to COVID-19 and reduce inflammation (Iacobellis, 2020), which has been shown to provide cardiovascular and cognitive benefits to the population.

### 3.3 Ketogenic diet

The ketogenic diet (KD) is characterized by very low-carbohydrate and high-fat, which induces a metabolic shift and elevates circulating ketone bodies (Ang et al., 2020). KD can mitigate the metabolic reprogramming and systemic inflammation in SARS-CoV-2 infection, including reduced serum proinflammatory cytokines storm (eg., TNF-α, IL-15, IL-22, G-CSF, M-CSF, MCP-1) and restored amino acid and energy metabolism (Palermo et al., 2023). Indirectly, KD was shown to shed weight along with glycemic control, which protected against oxidative stress in individuals with obesity and type 2 diabetes mellitus (T2DM). KD may be conducive to reducing ventilatory requirements, showing a potential as an adjuvant therapy for obese COVID-19 patients (Gangitano et al., 2021). KD leads to the polarization of M2 macrophages and the reduction of M1 macrophage development, which displays anti-inflammatory effects in COVID-19 patients (Sukkar and Bassetti, 2020).

The synthesis of ketone bodies including *β*-hydroxybutyrate (BHB), and the immune function of CD4^+^ T cells are impaired in SARS-CoV-2-induced ARDS. BHB, as an alternative carbon source, promoted oxidative phosphorylation and produced bioenergy amino acids and glutathione, thus remodeling the mitochondrial reoxidation-reduction balance of CD4^+^ T cells and restoring the immune function of CD4^+^ T cells. KD or BHB supplementation restored mitochondrial metabolism and immune function of CD4^+^ T cells, thereby reducing severe illness and mortality in mice during severe SARS-CoV-2 infection (Karagiannis et al., 2022). SARS-CoV-2 encodes viroporines E and 3a to activate the NOD-like receptor family pyrin domain containing 3 (NLRP3) inflammasome, which could be inhibited by BHB through the reduction of K^+^ efflux from macrophages and the secretion of IL-1β and IL-18 secretion (Paoli et al., 2020). Consistently, KD was demonstrated to restrain aging-facilitated coronavirus infection in mice, showing activation of ketogenesis, enriched γδ T cells, reduced NLRP3 inflammasome and decreased pathogenic monocytes in the lungs (Ryu et al., 2021). Reduced muscle mass was shown to be related to the onset of complications from COVID-19, and KD represents a viable approach to preserving muscle mass in post-COVID (Schiaffino et al., 2021). Thus, healthy gut microbiota and diet-derived metabolites may enhance antiviral defenses, while the inflammatory metabolism may exacerbate COVID-19 and hinder the recovery of post-COVID-19.

## 4 Interventions mediated by gut microbiota for COVID-19 and PACS

### 4.1 Dietary supplements

Several countries have compiled guidelines for dietary recommendations by healthy organizations during the COVID-19 pandemic, which are mostly in agreement. They advocate the intake of whole grains, fruits, and vegetables, which supply essential vitamins, minerals and hydration (de Faria Coelho-Ravagnani et al., 2021). Fruits and vegetables may reduce the risk of high blood pressure, diabetes and obesity, all of which are important risk factors for COVID-19 complications (Maggini et al., 2018; Wang et al., 2020). A recent study indicated the antiviral effects of luteolin against SARS-CoV-2, which could be enhanced by vitamin C, magnesium and zinc (Ferreira et al., 2024). Vitamins A, B2, C, D, and E could modulate the microbial diversity and composition as well as increase the production of SCFAs in varying degrees. A dietary supplement of vitamin B2 for 14 days increased the number of butyrate producers, namely *F. prausnitzii and Roseburia* (Pham et al., 2021). Vitamin E supplementation enhanced the synthesis of SCFAs and the proportion of beneficial bacteria such as *Akkermansia*, *Lactobacillus*, *Bifidobacterium*, and *Faecalibacterium* (Choi et al., 2020). SCFAs reduce viral loads in the respiratory tract and gastrointestinal tract by suppressing the expression of the SARS-CoV-2 receptor ACE2 and boosting adaptive immunity (Brown et al., 2022). COVID-19 patients displayed impaired SCFA and L-isoleucine biosynthesis in their gut microbiome that persisted beyond 30 days post-recovery and was associated with the severity of disease and host immune responses. Strategies to supplement SCFA or L-isoleucine might be formulated to promote the prognosis of COVID-19 (Zhang F. et al., 2022).

The issuance of some guidelines on the management of long-term COVID guides certain appropriate patient rehabilitation but awaits clinical practice and individualized strategy (Koc et al., 2022). Dietary multivitamin supplements were demonstrated to improve the clinical symptoms of long COVID (Nurek et al., 2021). Diets rich in omega-3 fatty acids and low in trans fats and refined carbohydrates enhance mental health and aid in the recovery from PACS. Whether an anti-inflammatory diet can dysregulate immune response and be extended to PACS patients is currently being studied by clinical trials (Wang C. et al., 2023). Dietary supplements such as acetyl-L-carnitine, hydroxytyrosol, and vitamins B, C, and D have been shown to have significant potential in improving PACS characterized by chronic fatigue (Naureen et al., 2021). Furthermore, mast cell activation and histamine release may play a role in acute COVID-19 infection and chronic PACS, indicating a therapeutic and prognostic potential for an anti-histamine diet (Afrin et al., 2020). Histamine intolerance in PACS may be related to a reduction in diamine oxidase levels, resulting in mast cell activation syndrome. Dietary polysaccharides were excavated as promising management for PACS with multiple biological activities, including immunomodulatory, antioxidant, and antiviral activities (Cheong et al., 2023). A clinical study showed that fermented tropical fruits such as *Morinda citrifolia* and *Carica papaya* diminished the deteriorated heart and lung functions manifestations of long-COVID, via restoring the immune cells, generating energy, suppressing inflammation and balancing redox status (Kharaeva et al., 2022). Two trials examining the effects of nicotinamide riboside and low-dose naltrexone (LDN) are currently underway (NCT04809974, NCT04604704) to reduce cognitive symptoms and fatigue by regulating inflammatory responses (Omran and Almaliki, 2020). A healthy diet could be considered a safe and cost-effective way to improve post-COVID symptoms and possible future complications.

### 4.2 Probiotics

The supplement of probiotics has been suggested as an auxiliary treatment for COVID-19 (Conte and Toraldo, 2020; Stavropoulou and Bezirtzoglou, 2020; Patra et al., 2021; Santacroce et al., 2021). Probiotics may favor virus clearance by enhancing immune function, inhibiting the activation of NLRP3 inflammasome (Synodinou et al., 2022), protecting the gut barrier, and preventing the co-infection of COVID-19 related bacteria (Patra et al., 2021; Bottari et al., 2021). Several clinical trials have been initiated. Probiotics formulation (*S. thermophilus DSM 32345, L. acidophilus* DSM 32241*, L. helveticus* DSM 32242*, L. paracasei* DSM 32243*, L. plantarum* DSM 32244*, L. brevis* DSM 27961*, B. lactis* DSM 32246*, B. lactis* DSM 32247) supplementation to patients with COVID-19 could effectively alleviate diarrhea, fever, asthenia, headache and other symptoms. The risk of respiratory failure was estimated to be 8 times lower in patients treated with bacteriotherapy than in the control group (d'Ettorre et al., 2020). A single-center, quadruple-blinded randomized controlled trial evaluating the efficacy and safety of a probiotic formulation consisting of three strains of *Lactiplantibacillus plantarum* and *Pediococcus acidilactici* in symptomatic COVID-19 outpatients, showed significantly reduced viral load and lung abnormality scores and increased SARS-CoV-2 specific antibodies (Gutiérrez-Castrellón et al., 2022). Similar benefits were also observed in hospitalized patients, probiotics containing *Lactobacillus* and *Bifidobacterium* strains reduced overall symptoms and decreased hospitalization duration and recovery time, which suggested the potential of probiotics in COVID-19 (Xu et al., 2022). *Lactobacilli* may reduce the risk of gastrointestinal symptoms, respiratory failure and death by regulating the immune response, reducing inflammation, and exerting direct antiviral effects (Taufer et al., 2024).

Probiotics can not only resist the virus but also improve the effectiveness of the vaccine. The probiotic *Lactobacillus plantarum* GUANKE after SARS-CoV-2 vaccination was able to boost both effective humoral and cellular responses by enhancing interferon signaling and inhibiting apoptotic and inflammatory pathways against COVID-19 in mice. However, in another randomized, controlled, single-center, open-label trial (NCT04854941), probiotics (*Lacticaseibacillus rhamnosus* PDV 1705, *B. bifidum* PDV 0903, *Bifidobacterium longum* subsp. *infantis* PDV 1911, and *B. longum* subsp. *longum* PDV 2301) were effective in treating COVID-19 related diarrhea, but did not significantly reduce mortality (Ivashkin et al., 2023). Therefore, more investigations are warranted to investigate the safety and efficacy of probiotics in COVID-19 patients.

Recently, a synbiotic preparation (SIM01), which includes three bacterial strains (*B. adolescentis*, *B. bifidum*, and *B. longum*), has been demonstrated to hasten the SARS-CoV-2 antibody formation, reduce the nasopharyngeal viral load and pro-inflammatory markers, and restore gut dysbiosis in patients with COVID-19 (Zhang L. et al., 2022). Surprisingly, SIM01 was found to significantly alleviate multiple symptoms of PACS at 6 months after acute infection compared to a placebo, accompanied by beneficial changes in the gut microbiota (Lau et al., 2024a). A clinical trial is underway to evaluate the effectiveness of probiotic and prebiotics supplements in normalizing the gut microbiota composition and immune function in long-COVID (NCT04813718) (Crook et al., 2021). The supplementation with probiotics during hospitalization may prevent the development of chronic fatigue of COVID-19 by impacting metabolites involved in glucose utilization and energy metabolism, including increased levels of Arginine, Asparagine, Lactate and reduced levels of 3-Hydroxyisobutirate (Santinelli et al., 2021). The study provides a new perspective for treating post-acute COVID-19 syndrome by regulating the gut microbiota.

### 4.3 Prebiotics

Prebiotics are non-digestible food components that promote the growth and activity of beneficial bacteria in the colon through fermentation (Salminen et al., 2021). By serving as substrates for probiotics, prebiotics suppress the proliferation of harmful bacteria and enhance the breakdown and assimilation of vital nutrients (Markowiak and Śliżewska, 2017). One study showed that inulin treatment increased the abundance of *Bifidobacterium* and *Lactobacillus*, thereby increasing SCFA production and thus regulating the gut microbiome (Vandeputte et al., 2017). Pectin and 1-kestose may induce the proliferation of *F. prausnitzii* with anti-inflammatory effects (Tochio et al., 2018). Phenolic compounds are also considered potential prebiotics as they interact with transcription factors such as NF-κB and Nrf2 exerting immunomodulatory effects. A randomized controlled trial showed that oral administration of curcumin significantly reduced morbidity, mortality and recovery time in patients with mild, moderate, and severe COVID-19 symptoms (Ahmadi et al., 2021; Batista et al., 2023). The metabolic product of *Lactiplantibacillus plantarum*, plantaricin BN, D, W, and JLA-9, presented antiviral activity by blocking the essential protein S during the SARS-CoV-2 life cycle (Anwar et al., 2021). In addition, the most common side effects of prebiotics are abdominal bloating and discomfort when consumed in large quantities (Hu et al., 2021). More researches on postbiotics and SARS-CoV-2 infection are needed, especially considering animal and human populations, to develop well-planned clinical trials and establish sufficient parameters to draw safe scientific conclusions and optimal dosage. Similarly, combined co-supplementation of both probiotics and prebiotics, in the form of synbiotics such as the blend of the *Lactobacillus* strain with inulin has been shown to improve health outcomes of individuals with long COVID, leading to reduced cough, fatigue and gut symptoms.

### 4.4 Fecal microbiota transplantation

FMT is a treatment where fecal bacteria from healthy donors or complex microbial communities from *in vitro* culture or purification of fecal materials are inoculated into the intestines of patients. FMT was attested to partially restore gut dysbiosis, alleviate gastrointestinal symptoms, and modulate the immune status of peripheral lymphocyte subsets in discharged COVID-19 patients (Liu et al., 2021). FMT restores the imbalanced gut microbiome and may affect immune responses including the respiratory system through the gut-lung axis, which may contribute to pulmonary-epithelial resistance to SARS-COV-2. The application of FMT in COVID-19 is still in the early stages of research. Most studies related to FMT and COVID-19 focus on general microbiome modulation rather than specific donor criteria. Currently, there is limited detailed literature regarding donor selection for FMT in the context of COVID-19 prevention and treatment. Recently, Biliński *et al.* have been performing a clinical trial (NCT04824222) that attempted to validate the effectiveness of FMT with the progression of COVID-19 and its association with cytokine storms and inflammation escalation (FeMToCOVID). As the first clinical trial using FMT in COVID-19, FeMToCOVID inhibits the inflammatory process by reconstructing the right influence of the gut-lung axis could be fundamental meaning to the development of ARDS in COVID-19 patients. Notably, the gut microbiome of donors recovered from COVID-19 may be altered and miss critical microbiome, which compromises the efficacy of FMT for COVID-19 (Kazemian et al., 2021).

A pilot study containing 60 PACS patients with insomnia showed that FMT could be effective and safe in alleviating sleep disturbance in PACS, with the enrichment of bacteria such as *Gemmiger formicilis* and depletion of menaquinol derivatives production (NCT05556733) (Lau et al., 2024b). To prevent the transmission of SARS-CoV-2 in FMT, donors should be screened for travel history and symptoms of COVID-19, as well as exposure of patients before each stool donation. Donors who pass this screening should be tested for COVID-19 while those who fail are excluded from fecal donation (Khanna and Pardi, 2020). There is an urgent need for comprehensive and optimized fecal donor screening programs to ensure the safety and effectiveness of FMT during the COVID-19 pandemic. Uncovering the bidirectional effects of the gut-lung axis is critical to adapt traditional FMT strategies and novel microbiome-based therapeutic interventions for COVID-19 in the future.

## 5 Conclusions and prospects

SARS-CoV-2, as a highly infectious respiratory virus, induced the ongoing COVID-19 global pandemic. Even with the evolution of preventive vaccines and medicine, persistent and prolonged effects after acute COVID-19 have debilitated millions of subjects worldwide. SARS-CoV-2 infection could impact the complex gut community and drive immunological changes. Conversely, the alterations of gut microbiota have been demonstrated to have long-reaching and long-lasting potential in COVID-19 and PACS, which remains unexplored. The heterogeneity and complexity of PACS hindered the decryption of the pathophysiological mechanisms, which need further multidisciplinary studies to better understand and address these underlying health problems. Some predicting models have been used to identify individuals with a high risk of long-COVID to process education and rehabilitation in the early phase (Sudre et al., 2021). The involvement of coexisting symptoms suggests the requires to provide insights into different symptom clusters and identify potential targets for personalized therapeutic interventions. Current treatment options for PACS still primarily emphasize managing the symptoms but neglect the underlying cause, such as persistent inflammation, immune dysfunction, and tissue damage.

Multiple risk factors may predispose patients to worse consequences in acute infection and promote PACS through microbial and immune regulation. This review emphasized the gut microbiota dysbiosis in COVID-19 and the sequelae of PACS. Elucidating the underlying mechanisms may contribute to propose personalized and non-invasive strategies for patients with severe disease progression or complications post-acute infection. However, prospective studies are required to determine whether gut microbiota alterations contribute to or result from SARS-CoV-2 infection, and the effects of the drugs, especially the antibiotics in COVID-19 cannot be ignored. An incorporated dietary therapy or specialized diet, as one of the modifiable factors, is more inclined for patients to facilitate post-COVID-19 recovery. Thus, subjects should be mindful of dietary habits to reduce susceptibility to and long-term sequelae from COVID-19. Personalized nutrition which modulates the gut microbiota might be a crucial prophylactic factor to minimize the disturbance on COVID-19 patients. Reducing the risk of dietary habits in every phase of COVID-19 will play a crucial role in combatting its pandemic and postinfection effect with minimum financial loss and adverse outcomes. Regrettably, clinical outcomes of diet supplements in long-COVID therapy are still lacking. Moreover, the review also highlights the possibility of targeting the gut microbiota as a conceivable therapeutic management for COVID-19 and PACS. The role of microbiome modulation strategies, such as healthy diet, probiotics or prebiotics, and FMT in regulating PACS warrants investigation.

## Glossary

ACE2: angiotensin-converting enzyme 2
ARDS: acute respiratory distress syndrome
BHB: *β*-hydroxybutyrate
CRP: C-reactive protein
COVID-19: coronavirus disease 2019
DALYs: disability-adjusted life years
FMT: fecal microbiota transplantation
GM-CSF: granulocyte-macrophage colony-stimulating factor
HFD: High-fat diet
HMGB1: high-mobility group box protein-1
IBD: inflammatory bowel diseases
IBS: irritable bowel syndrome
ICU: intensive care unit
KD: ketogenic diet
LBP: lipopolysaccharide-binding protein
LPS: lipopolysaccharide
MD: Mediterranean diet
NASH: non-alcoholic steatohepatitis
NLRP3: NOD-like receptor family pyrin domain containing 3
PACS: post-acute COVID-19 syndrome
PRRs: pattern-recognition receptors
SARS-CoV-2: severe acute respiratory syndrome coronavirus-2
SCFA: short-chain fatty acid
T2DM: type 2 diabetes mellitus
WD: Western diet
